# Clonal spread of trimethoprim-sulfamethoxazole–resistant Stenotrophomonas maltophilia isolates in a tertiary hospital

**DOI:** 10.3205/dgkh000481

**Published:** 2024-05-17

**Authors:** Ömür Mustafa Parkan, Hüseyin Kiliç, Emine Alp, Demet Timur, Aycan Gündoğdu, Özlem Ünaldi, Rıza Durmaz

**Affiliations:** 1 Department of Medical Microbiology, Faculty of Medicine, Erciyes University, Kayseri, Turkey; 2 Department of Infectious Diseases and Clinical Microbiology, Faculty of Medicine, Ankara Yildirim Beyazit University, Ankara, Turkey; 3 Department of Medical Microbiology, Bursa City Hospital, Bursa, Turkey; 4 National Molecular Microbiology Reference Laboratory, Public Health Institution of Turkey, Ankara, Turkey; 5 Department of Medical Microbiology, Faculty of Medicine, Ankara Yildirim Beyazit University, Ankara, Turkey

**Keywords:** Stenotrophomonas maltophilia, trimethoprim-sulfamethoxazole resistance, PFGE, sul, integron

## Abstract

**Aim:**

The aims of this study were to: (i) determine antibiotic susceptibility of clinical *Stenotrophomonas maltophilia* isolates, (ii) investigate the presence of different classes of integrons and *sul* genes responsible for sulphonamide resistance, (iii) assess the molecular epidemiology of the isolates by determining their clonal relatedness, and (iv) investigate the potential sources of infection by collecting environmental samples when necessary.

**Methods:**

99 *S. maltophilia* isolates from clinical specimens of hospitalized patients were screened by PCR for *sul1*, *sul2*, *sul3* genes, and integron-associated integrase genes: *intI1*, *intI2*, and *intI3*. PFGE was used to determine the clonal relatedness of the isolates.

**Results:**

Susceptibility rates for trimethoprim-sulfamethoxazole, levofloxacin, and ceftazidime were 90.9%, 91.9%, and 53.5% respectively. All trimethoprim-sulfamethoxazole–resistant isolates were positive for *intI1* and *sul1*. PFGE analysis revealed that 24 of the isolates were clonally related, clustering in seven different clones. Five of the nine trimethoprim-sulfamethoxazole–resistant isolates were clonally related. The first isolate in this clone was from a wound sample of a patient in the infectious diseases clinic, and the other four were isolated from the bronchoalveolar lavage samples of patients in the thoracic surgery unit. The patient with the first isolate neither underwent bronchoscopy nor stayed in the thoracic surgery unit. Although clustering was observed in bronchoalveolar lavage samples, no *S. maltophilia* growth was detected in environmental samples.

**Conclusion:**

The findings demonstrated that the *sul1* gene carried by class 1 integrons plays an important role in trimethoprim-sulfamethoxazole resistance in *S. maltophilia* isolates. PFGE analysis revealed a high degree of genetic diversity. However, detection of clonally related isolates suggests the acquisition from a common source and/or cross-transmission of this microorganism between the patients.

## Zusammenfassung

**Zielsetzung:**

In dieser Studie sollten (i) die Antibiotika-Empfindlichkeit klinischer *Stenotrophomonas maltophilia-*Isolate bestimmt, (ii) das Vorhandensein verschiedener, für die Sulfonamid-Resistenz verantwortlichen Klassen von Integronen und *sul*-Genen, untersucht, iii) die molekulare Epidemiologie der Isolate durch Bestimmung ihrer klonalen Verwandtschaft bewertet und (iv) potenzielle Infektionsquellen durch Umgebungsuntersuchungen überprüft werden.

**Methoden:**

99 *S. maltophilia-*Isolate aus klinischen Proben von Krankenhauspatienten wurden mittels PCR auf die Gene *sul1*, *sul2* und *sul3* sowie die Integron-assoziierten Integrase-Gene *intI1*, *intI2* und *intI3* untersucht. Mit PFGE wurde die klonale Verwandtschaft der Isolate bestimmt.

**Ergebnisse:**

Die Empfindlichkeitsraten für Trimethoprim-Sulfamethoxazol, Levofloxacin und Ceftazidim betrugen 90,9%, 91,9% bzw. 53,5%. Alle Trimethoprim-Sulfamethoxazol-resistenten Isolate waren positiv für *intI1* und *sul1*. Die PFGE-Analyse ergab, dass 24 der Isolate klonal verwandt waren und sich in sieben verschiedenen Klonen zusammenschlossen. Fünf der neun Trimethoprim-Sulfamethoxazol-resistenten Isolate waren klonal verwandt. Das erste Isolat dieses Klons stammte aus der Wunde eines Patienten in der Klinik für Infektionskrankheiten, die anderen vier wurden aus bronchoalveolären Lavageproben von Patienten in der Thoraxchirurgie isoliert. Der Patient mit dem ersten Isolat unterzog sich weder einer Bronchoskopie noch hielt er sich in der Abteilung für Thoraxchirurgie auf. Obwohl in den bronchoalveolären Lavageproben eine Clusterbildung beobachtet wurde, war *S. maltophilia* nicht in den Umgebungsuntersuchungen nachweisbar.

**Fazit:**

Die Ergebnisse zeigen, dass das von Klasse-1-Integronen getragene *sul1*-Gen eine wichtige Rolle bei der Trimethoprim-Sulfamethoxazol-Resistenz von *S. maltophilia-*Isolaten spielt. Die PFGE-Analyse ergab ein hohes Maß an genetischer Vielfalt. Der Nachweis klonal verwandter Isolate deutet jedoch darauf hin, dass dieser Mikroorganismus aus einer gemeinsamen Quelle stammt und/oder zwischen den Patienten übertragen wird.

## Introduction

*Stenotrophomonas maltophilia* is a ubiquitous microorganism commonly found in nature and hospitals, especially in humid environments [1]. It is difficult to treat infections caused by *S. maltophilia* because of its hereditary resistance to many antibiotics. It can also acquire new resistance mechanisms through horizontal gene transfer or mutations [2]. Trimethoprim-sulfamethoxazole (TMP/-SMX) is the first antimicrobial agent of choice in treatment; however, studies conducted worldwide have reported varying rates of resistance to this antibiotic [3], [4]. The *sul1* gene carried by class 1 integrons and the *sul2* gene associated with insertion sequence common region (IS*C*R) elements were found to be associated with TMP/SMX resistance in *S. maltophilia*; surveillance studies are recommended since TMP/SMX resistance has the potential to increase through mobile genetic elements [5]. As they cause problems in treatment, epidemiological typing of nosocomial pathogens, especially multi-resistant microorganisms, allows monitoring of the spread of these agents [6]. In addition to determining antibiotic resistance patterns for the control of nosocomial infections due to *S. maltophilia*, typing of isolates with molecular epidemiological studies is also important [7]. This study aimed to: (i) determine the susceptibility of clinical *S. maltophilia* isolates to TMP/SMX, levofloxacin, and ceftazidime, (ii) investigate the presence of integrons and *sul* genes, (iii) evaluate the molecular epidemiology by using pulsed-field gel electrophoresis (PFGE), and (iv) investigate the potential sources of infection by collecting environmental samples when necessary.

## Methods

### Bacterial isolates

*S. maltophilia* isolates from clinical specimens of patients in Erciyes University Faculty of Medicine Hospitals over a 16-month period were evaluated. Clinical specimens were inoculated on BD Columbia agar with 5% sheep blood (BBL™, Becton Dickinson, Heidelberg, Germany) and eosin-methylene blue (EMB) agar (Oxoid, Basingstoke, UK) for culture. The blood culture samples were incubated in the BacT/ALERT^®^ 3D automated blood culture system (bioMérieux, Durham, NC, USA). The bottles with a positive signal were subcultured on BD Columbia agar with 5% sheep blood and EMB agar. Environmental samples were incubated in tryptic soy broth (Oxoid Ltd., Basingstoke, UK) at 37° C for 48 hours before they were subcultured on BD Columbia agar with 5% sheep blood and EMB agar. The BD Phoenix™ automated identification system (Becton Dickinson, Sparks, MD, USA) was used to identify the isolated microorganisms. A single *S. maltophilia* isolate from each patient was included in the study. 

### Antimicrobial susceptibility tests

The broth microdilution method was used for ceftazidime and levofloxacin, and the Etest (gradient diffusion) method was used for TMP/SMX to determine the susceptibility of *S. maltophilia* isolates. Clinical and Laboratory Standards Institute (CLSI) criteria were used to evaluate the results [8]. 

### Broth microdilution

The broth microdilution method was performed by preparing two-fold concentrations of antibiotics in cation-adjusted Mueller-Hinton broth (Oxoid, Basingstoke, UK) in accordance with CLSI recommendations [8]. The bacterial suspension was inoculated with a final inoculum concentration of 5×10^5^ CFU/mL. *Pseudomonas aeruginosa* strain ATCC^®^ 27853 was used for quality control. After 20 hours of incubation at 35°C, the lowest antibiotic concentration with no visible growth was determined as the minimal inhibitory concentration (MIC). 

### Etest (gradient diffusion)

This was carried out on cation-adjusted Mueller Hinton agar (Merck, Darmstadt, Germany) medium in accordance with the recommendations of the manufacturer (bioMérieux, Marcy l’Etoile, France). *Escherichia coli* strain ATCC^®^ 25922 was used for quality control. 

### Investigation of integron-associated intI and sul genes

DNA was extracted from the isolates grown in pure culture, using the PureLink™ Genomic DNA Mini Kit (Invitrogen, Carlsbad, CA, USA) in accordance with the manufacturer’s recommendations. The primer pairs described previously were used for PCR and are shown in Table 1 (Tab. 1) [9], [10], [11], [12], [13]. AmpliTaq Gold^®^ 360 Master Mix (Applied Biosystems, Foster City, CA, USA) and Labcycler instrument (SensoQuest, Göttingen, Germany) were used for PCR. 

### Integron-associated intI1, intI2, and intI3 genes

All isolates were tested for integron-associated integrase genes for three different classes of integrons according to the following PCR protocol: an initial denaturation of 94°C for 4 min, followed by 35 cycles comprising a denaturation step of 94°C for 1 min, an annealing step of 56°C for 1 min, and an extension step of 72°C for 1 min. This cycling step was followed by a final extension time of 72°C for 10 min. 

### Sul1 and sul2 genes

All isolates were tested for *sul1* and *sul2* genes according to the following PCR protocol: initial denaturation of 95°C for 6 min, followed by 37 cycles consisting of a denaturation step of 95°C for 30 s, an annealing step of 62°C for 45 s, and an extension step of 72°C for 1 min. This cycling step was followed by a final extension time of 72°C for 7 min. 

### Sul3 gene

All isolates were tested for *sul3* gene according to the following PCR protocol: an initial denaturation of 95°C for 5 min, followed by 35 cycles comprising a denaturation step of 95°C for 40 s, an annealing step of 62°C for 40 s, and an extension step of 72°C for 1 min. This cycling step was followed by a final extension time of 72°C for 5 min.

### Sequence analysis of the PCR products

PCR products were purified by using ExoSAP-IT^®^ PCR Product Cleanup Reagent (Affymetrix, Santa Clara, CA, USA). The BigDye^®^ Terminator v3.1 Cycle Sequencing Kit (Applied Biosystems, Foster City, CA, USA) was used in the cyclic sequencing reaction of purified PCR products. Two separate cyclic sequencings were performed according to the manufacturer’s recommendations, using forward and reverse primers for each sample. After the cyclic sequencing reaction, precipitation was performed with 2 µL of 125 mM EDTA, 2 µL of 3 M sodium acetate, and 50 µl of 100% ethanol. Samples were sequenced using an ABI 3500xL Genetic Analyzer (Applied Biosystems, Foster City, CA, USA). The DNA sequences were evaluated by comparing them with sequences in the National Center for Biotechnology Information (NCBI) database using the BLAST^®^ sequence analysis tool (http://blast.ncbi.nlm.nih.gov/Blast.cgi).

### PFGE analysis

PFGE was carried out as previously described, with some modifications [14]. Bacterial suspensions equivalent to McFarland standard turbidity 4 in cell suspension buffer (100 mM Tris, 100 mM EDTA, pH 8.0) were prepared from pure cultures. 1,000 µL of the bacterial suspensions were centrifuged at 15,000×g for 2 min. After discharging the supernatant, 1,000 µL of cell suspension buffer and 25 µL of proteinase K were added to the pellet and mixed. Bacterial suspensions were mixed with 1% low melting point agarose (Bio-Rad Laboratories, Hercules, CA, USA) containing 1% sodium dodecyl sulfate and filled into the plug molds. The solidified agarose plugs were placed in tubes containing 5 mL of cell lysis buffer (50 mM Tris, 50 mM EDTA, pH 8.0, 1% sarcosine) and 25 µL proteinase K and kept in a shaking water bath set at 55°C for 2 h. After lysis, the buffer was discharged and the tubes were filled with 10 mL of sterile ultrapure water, then washed for 15 min in a shaking water bath at 55°C. The same procedure was performed once again with ultrapure water, then repeated three times using TE buffer (10 mM Tris, 1 mM EDTA, pH 8.0). A piece measuring 4×5.5 mm was cut from each plug. The cut pieces were placed in microcentrifuge tubes containing 100 µL of restriction buffer (FastDigest^®^, Fermentas, Vilnius, Lithuania) and incubated at 37°C for 10 min. After the restriction buffer in the tubes was removed with a micropipette, 2.5 µL of restriction enzyme SpeI (FastDigest^®^, Fermentas, Vilnius, Lithuania), 10 µL of restriction buffer, and 87.5 µL of nuclease-free sterile distilled water were added to each tube and incubated at 37°C for 1 h. For electrophoresis, 1% PFGE-grade agarose (Amresco, Solon, OH, USA) was prepared and the CHEF-DRII system (Bio-Rad Laboratories, Nazareth, Belgium) was used. The running temperature was set at 14°C under the following conditions: switch times ranging from 5 to 30 s, angle 120°, field strength 6 V/cm for 16 h; and switch times ranging from 1 to 5 s, angle 120°, field strength 6 V/cm for 4 h. Gel Logic 2200 Imaging System (Kodak, Rochester, NY, USA) was used to photograph the DNA bands. The images were analyzed using BioNumerics version 7.5 (Applied Maths, Sint-Martens-Latem, Belgium). Cluster analysis was performed using the unweighted pair group method with mathematical averaging (UPGMA) algorithm, and the molecular relatedness of the isolates was determined using the Dice coefficient, with 1.5% tolerance and 1% optimization. Isolates with ≥90% similarity were considered to be in the same clone and were named with a letter.

### Statistical analysis

Fisher’s exact test was used to evaluate differences in the prevalence of intI between antibiotic-resistant isolates. A *p*-value less than 0.05 was considered statistically significant.

## Results

### S. maltophilia isolates

A total of 99 *S. maltophilia* isolates from various clinical specimens were included in the study. 52.5% of the patients were male; the median age was 59 (range: 0–89). While 47.5% of the patients were hospitalized in intensive care units (ICUs), 35.4% were hospitalized in non-surgical clinics (14.2% hematology and oncology, 7.1% pulmonology, 5.1% infectious diseases, 9% other), and 17.1% in surgical clinics (14.1% thoracic surgery, 2% general surgery, 1% gynecology). 46.5% of the clinical samples were respiratory tract samples [24.2% endotracheal aspirate (ETA), 17.2% bronchoalveolar lavage fluid (BALF), 5.1% sputum]. This was followed by blood (28.3%), wound (10.1%), pleural fluid (7.1%), drainage fluid (3%), abscess (2%), urine (1%), peritoneal fluid (1%) and bile (1%).

### Antimicrobial susceptibility profiles

Of the 99 *S. maltophilia* isolates, 90.9% were susceptible to TMP/SMX, 91.9% to levofloxacin, and 53.5% to ceftazidime. The susceptibility of the isolates to antibiotics is shown in Table 2 (Tab. 2).

### Presence of integron-associated intI1, intI2, and intI3

In nine isolates (9.1%), *intI1* was detected by PCR. All isolates were negative for *intI2* and *intI3*. When evaluated together with the antibiotic susceptibility results, it was found that the *intI1* gene was present in all TMP/SMX resistant isolates, and a statistically significant relationship was found between the presence of *intI1* and TMP/SMX resistance (*p*<0.001). No such relationship was found between ceftazidime and levofloxacin resistance and *intI1* positivity (Table 3 (Tab. 3)). 

### Presence of sul1, sul2, and sul3

In nine isolates (9.1%), *sul1* was detected by PCR. These nine isolates were found to be identical to the isolates with *intI1* and resistant to TMP/SMX. No isolates were positive for *sul2* and *sul3* by PCR.

### Sequence analysis of the PCR products

Sequence analysis of the PCR products confirmed that the bands detected by PCR in nine isolates were *intI1* and *sul1*.

### PFGE analysis

According to PFGE analysis, 75 of the isolates were sporadic and 24 were found to be clonally related (similarity ≥90%). The clonally related isolates were collected in seven different clones (A–G), each containing 2–7 isolates (Table 4 (Tab. 4)).

### Environmental samples

Environmental samples were collected from fiberbronchoscopes and washer-disinfectors, due to the detection of clustering in BALF samples in PFGE analysis. There was no growth of *S. maltophilia* in the cultures. However, *P. aeruginosa*, *Pseudomonas putida*, and *Enterococcus faecium* were detected in the channel of one of the bronchoscopes; *Achromobacter xylosoxidans* was detected on the inner surface and in the rinse water of the washer-disinfector. No microorganism growth was observed in the samples taken after appropriate cleaning and disinfection processes. 

## Discussion

*S. maltophilia* is a multi-drug-resistant microorganism that can be isolated from many water-related environmental sources inside and outside hospitals and causes nosocomial infections with an increasing incidence [1]. *S. maltophilia* infections are more common in patients in ICUs than in the general population [4]. In a study evaluating *S. maltophilia* isolates collected from 24 participating university hospitals in Europe, it was reported that 41.5% of the isolates were obtained from ICU patients [15]. It has been reported that this rate can reach up to 70% [16]. In this study, 47.5% of the isolates were from patients hospitalized in ICUs. This can be explained by the fact that ICU patients have many risk factors reported for *S. maltophilia* infections, such as immunodeficiency, malignancy, chronic lung diseases, mechanical ventilation, indwelling catheters and similar devices, as well as the use of broad-spectrum antibiotics [17].

*S. maltophilia* is mainly associated with respiratory tract infections [17]. Among the pneumonia agents isolated from invasive respiratory tract specimens of patients hospitalized in 53 centers in the USA and Europe, *S. maltophilia* was the sixth and ninth most frequently isolated microorganism, respectively [18]. Gulmez and Hascelik [7], in their study on 205 *S. maltophilia* isolates from 188 patients, isolated this microorganism with a frequency of 40%, 21.5%, and 13.2% from respiratory tract, blood, and pus samples, respectively. Juhasz et al. [16] reported that the most common sites of isolation for *S. maltophilia* in 100 patients with confirmed infection were ETA (31%), BALF (30%), blood (25%), and sputum (7%). In this study, *S. maltophilia* was most frequently isolated from respiratory tract samples (ETA 24.2%, BALF 17.2%, sputum 5.1%) and second-most frequently from blood samples (28.3%), which supports these findings.

The inherited resistance mechanisms of *S. maltophilia* to many antimicrobials cause problems in treatment. TMP/SMX is still accepted as the first-choice antimicrobial agent [3], [4], [17]. Analyzing the SENTRY Antimicrobial Surveillance Program data, Gales et al. [19] reported that the TMP/SMX susceptibility rates of 6467 *S. maltophilia* isolates collected globally between 1997–2016 showed little change over the years and was 95.6% overall. They reported the levofloxacin susceptibility as 81.5%. According to the data of the same surveillance program for the years 2014–2019, TMP/SMX, levofloxacin, and ceftazidime susceptibilities were reported as 95%, 79.6%, and 24%, respectively [20]. In comparison with these data, the susceptibility rate for TMP/SMX was lower (90.9%) and levofloxacin and ceftazidime susceptibility rates were higher (91.8% and 53.5%, respectively) in this study.

Determinating the resistance mechanisms of nosocomial pathogens and the identifying mobile genetic elements that play role in the transmission of resistance are important in terms of taking effective measures against these microorganisms. Most of the TMP/SMX resistance genes are located on transferable genetic elements. The sulfonamide resistance gene *sul1* and the *dfr* genes responsible for trimethoprim resistance are often associated with class 1 integrons; *sul2* and *sul3*, which are other sulfonamide resistance genes, were found to be mostly associated with plasmids and transposons [21]. Toleman et al. [5] investigated *intI1*, *sul1*, *sul2*, *sul3*, *dfr* genes, and IS*CR* elements in a total of 55 isolates, 25 of which were resistant to TMP/SMX and 30 of which were susceptible, collected from around the world, and detected *sul1* in association with class 1 integrons in 17 of the TMP/SMX resistant isolates. *sul2* was also shown in nine of the TMP/SMX resistant isolates, six of which were associated with IS*CR2*. Song et al. [22] tested 120 *S. maltophilia* isolates collected from three different university hospitals in Korea and found class 1 integrons in 17 of 28 *sul1* positive isolates. However, IS*CR2* and *sul2* were not detected, and TMP/SMX resistant isolates were reported to carry *sul1* and class 1 integrons significantly more frequently than did susceptible isolates. Kaur et al. [23] investigated *intI1*, *intI2*, *intI3*, *sul1*, and *sul2* in 106 clinical isolates, and *intI1* was detected in nine isolates, five of which were TMP/SMX resistant. They also reported that *intI2* and *intI3* were not found in any of the isolates. The co-existence of *sul1* and *sul2* genes was reported in two of the TMP/SMX resistant isolates, while the remaining 22 isolates carried either *sul1* (10 isolates) or *sul2* (12 isolates). In our study, *intI1* and *sul1* were detected together in all nine TMP/SMX resistant isolates, and these genes were not found in susceptible isolates. TMP/SMX MIC was >32/608 µg/mL for eight of the nine isolates, and 8/152 µg/mL for the remaining isolate. A significant relationship was found between TMP/SMX resistance and the presence of *intI1*, but a similar relationship could not be shown for ceftazidime and levofloxacin resistance. These results, in line with the findings of other researchers, indicate that the *sul1* gene located in the 3'-conserved region of class 1 integrons has an important role in TMP/SMX resistance in *S. maltophilia* isolates. This situation poses a threat to the spread of resistance to TMP/SMX as a result of horizontal gene transfer and underlines that TMP/SMX resistance should be monitored together with its molecular mechanisms. 

The problems arising in the treatment of *S. maltophilia* infections due to resistance problems increase the importance of epidemiological characterization of clinical strains. Various molecular typing methods, especially PFGE, were used to investigate the epidemiology of infections and nosocomial outbreaks due to this microorganism. In a study conducted in Spain, 132 clinical isolates from 105 hospitalized patients and seven environmental isolates were typed by using PFGE, and high genetic diversity was observed among isolates, despite originating from a single hospital. Five clones were identified that were claimed to be responsible for cross-transmission among patients, and it was noted that one *S. maltophilia* isolate from a fiberbronchoscope showed the same PFGE pattern as a clone containing respiratory isolates [24]. Neela et al. [25] typed 63 isolates in a tertiary hospital for one year and detected 59 different PFGE patterns. It was emphasized that there may be cross-transmission between wards due to the Isolation of strains with the same band profile from clinical samples of patients in different units. It was also underlined that continuous epidemiological monitoring is required besides antibiotic resistance surveillance, due to the potential for such spreads to occur with resistant isolates. Similarly, high genetic diversity was found among *S. maltophilia* isolates in our study and other studies using PFGE [26], [27]. This suggests the possibility that *S. maltophilia* may have been acquired from different sources as a result of its broad environmental distribution. Genetic diversity is also reported in studies using other molecular epidemiological methods, such as ERIC-PCR, rep-PCR, and MLST [7], [16], [28], [29], [30].

In this study, seven clones (A–G), each containing 2–7 isolates, were identified by PFGE analysis. This reveals the possibility of cross-transmission between patients or that these microorganisms were acquired from a common source, and underlines the importance of infection control measures in preventing the spread of multiresistant pathogens. This view was supported by studies reporting that the spread of *S. maltophilia* was limited with the help of the measures taken [31], [32], [33]. The increase in antibiotic resistance as a result of the clonal spread of resistant strains and subsequent treatment difficulties are serious problems in the treatment of infectious diseases. In this study, five of the nine TMP/SMX resistant isolates were in the same clone (clone E). The first isolate in this clone was from a wound sample of a patient in the infectious diseases clinic, and the other four were isolated from the bronchoalveolar lavage samples of patients in the thoracic surgery unit. The patient with the first isolate neither underwent bronchoscopy nor stayed in the thoracic surgery unit.

*S. maltophilia* can form biofilm and colonize medical instruments [34]. Cross-transmission and pseudoepidemics related to *S. maltophilia* have been reported in association with bronchoscopes [24], [35], [36]. In this study, clustering was observed in BALF samples according to PFGE results. The hospital infection control committee was informed and environmental samples were collected from fiberbronchoscopes and washer-disinfectors used by pulmonology and thoracic surgery units. However, there was no growth of *S. maltophilia* in the cultures. This situation might be due to the removal of *S. maltophilia* from the devices between the time of isolations from clinical samples and the time of environmental sampling. However, *P. aeruginosa*, *P. putida*, *A. xylosoxidans*, and *E. faecium* growth were detected in environmental cultures and it was decided to re-evaluate the disinfection process of the fiberbronchoscopes. In the following period, no bacterial growth was observed. This suggests that fiberbronchoscopes may also have been contaminated with *S. maltophilia*, resulting in pseudo-outbreaks, or infections in patients with predisposing factors.

## Conclusions

As an opportunistic pathogen, *S. maltophilia* can cause nosocomial infections, and its intrinsic antimicrobial resistance limits treatment options. Therefore, it is necessary to monitor the resistance of *S. maltophilia* to antimicrobials that can be used in treatment, especially TMP/SMX. The findings of our study demonstrated that the *sul1* gene, which is carried by class 1 integrons and therefore has the potential for horizontal transmission, plays an important role in TMP/SMX resistance in *S. maltophilia* isolates. In order to minimize the spread of resistant pathogens such as *S. maltophilia* in the hospital setting, there is a need for accurate and complete implementation of infection control measures and sterilization-disinfection procedures. Hence, molecular epidemiological studies are of great importance in the development of preventive strategies by investigating the transmission routes of nosocomial pathogens and in monitoring the effectiveness of infection control measures.

## Notes

### Funding

This study was supported financially by Erciyes University Scientific Research Projects Unit with the project code TTU-2015-5034.

### Ethics approval 

This study was performed in line with the principles of the Declaration of Helsinki. Approval was granted by the Ethics Committee of Erciyes University (07.02.2014/2014/74). Informed consent was obtained from all individual participants or their legal guardians.

### Authors’ ORCID

- Ömür Mustafa Parkan: 0000-0002-1071-4985
- Hüseyin Kiliç: 0000-0003-4885-4112
- Emine Alp: 0000-0003-0189-6008
- Demet Timur: 0000-0002-2475-5956
- Aycan Gündogdu: 0000-0003-2806-8464
- Özlem Ünaldi: 0000-0002-5560-6558
- Riza Durmaz: 0000-0001-6561-778X

### Competing interests

The authors declare that they have no competing interests.

## Figures and Tables

**Table 1:**
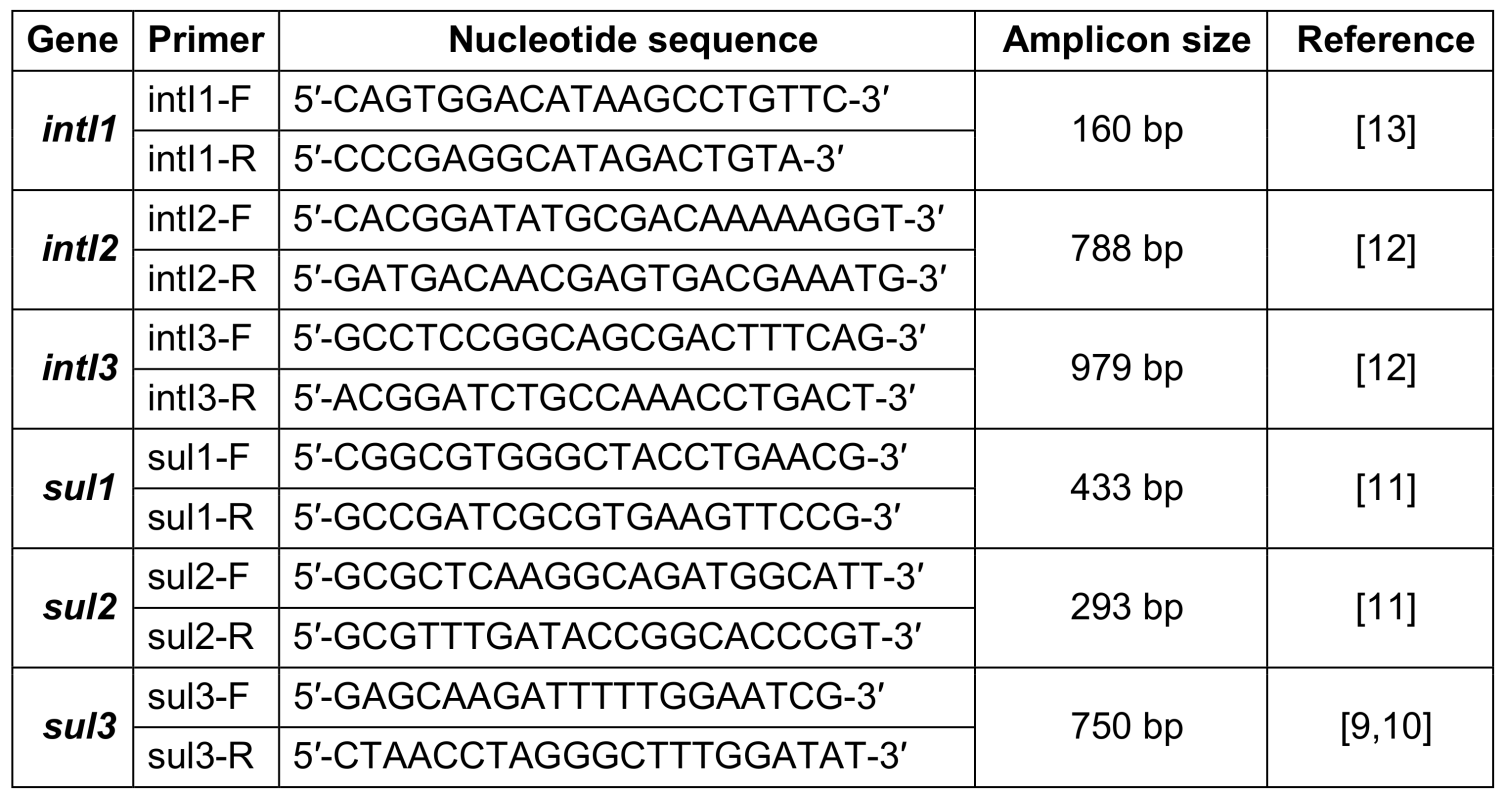
Primer pairs used in PCR

**Table 2:**
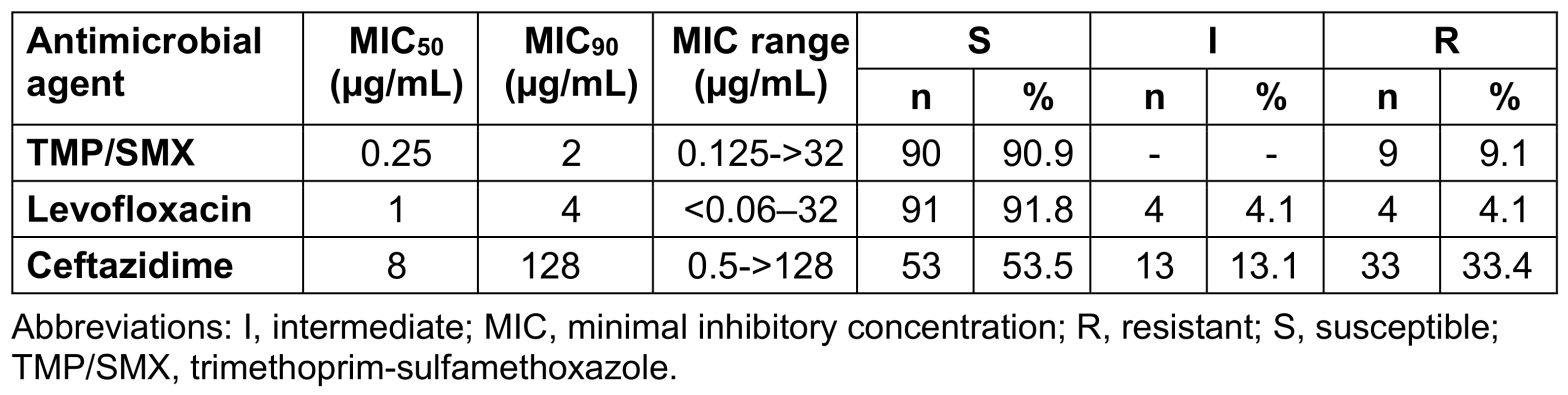
Activity of antimicrobial agents tested against 99 S*. maltophilia* isolates

**Table 3:**
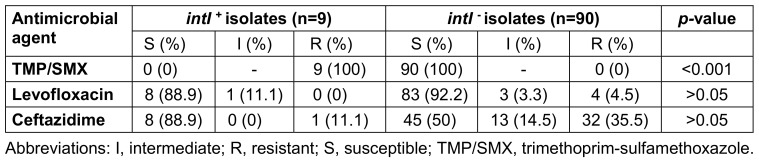
Prevalence of antimicrobial drug resistance among 99 *S. maltophilia* isolates with and without *intI* genes

**Table 4:**
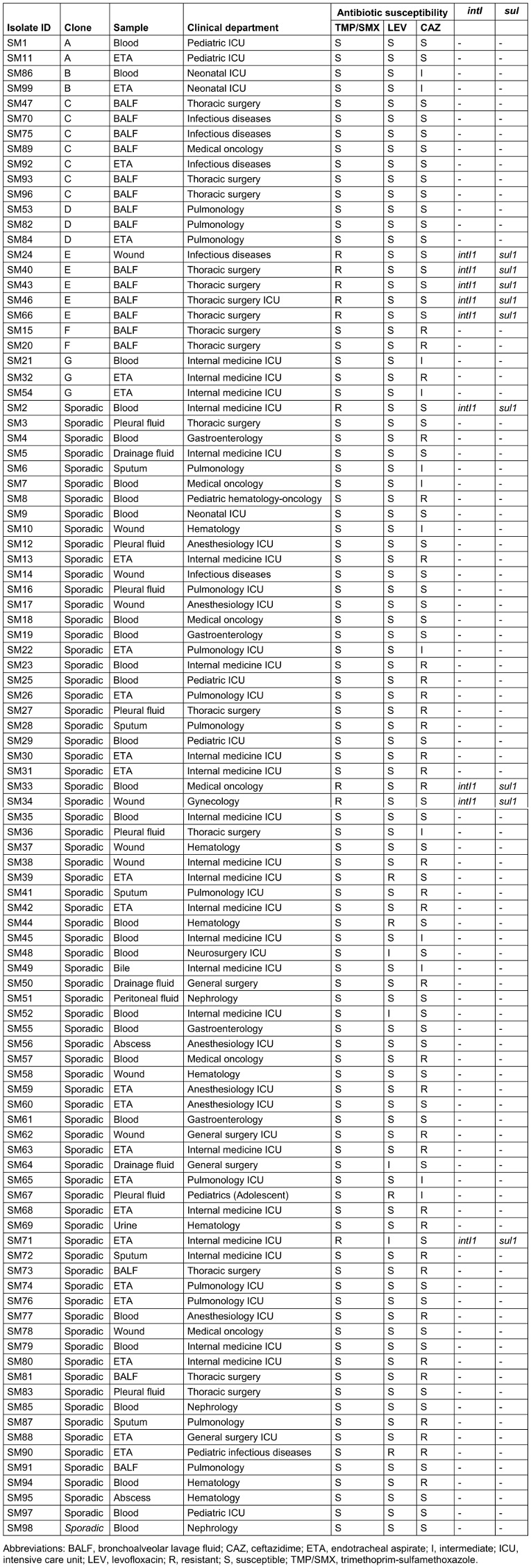
Clonal relatedness and antibiotic susceptibility of the isolates and presence of *intI* and *sul* genes
